# Performance and Perceptions of Health Care Professionals Using an Immersive Virtual Reality Tool for Home Care Training: Observational Feasibility and Acceptability Study

**DOI:** 10.2196/75104

**Published:** 2025-11-20

**Authors:** José Mira, Javier Rios, Eva Gil-Hernández, Nida Abed, Vanesa Ribeiro-Neves, Clara Pérez-Esteve, Mercedes Guilabert-Mora, Almudena Arroyo, Maria Purificacion Ballester

**Affiliations:** 1 Universitat de Miguel Hernández d'Elx Elche Spain; 2 Fundación para el Fomento de la Investigación Sanitaria y Biomédica de la Comunitat Valenciana Alicante Spain; 3 Hospital San Juan de Dios del Aljarafe Bormujos Spain; 4 Escola Paulista de Enfermagem-Unifesp Sao Paulo Brazil; 5 Fundación San Juan de Dios. Escuela Universitaria de Enfermería y Fisioterapia San Juan de Dios, Universidad Pontificia Comillas Bormujos Spain; 6 Pharmacology Department. Universidad Católica de San Antonio de Murcia Murcia Spain

**Keywords:** patient safety, virtual reality, augmented reality, health care providers, informal caregivers

## Abstract

**Background:**

Informal caregivers play a crucial role in home care and many lack formal training, potentially compromising patient safety. Immersive virtual reality (VR) offers an innovative approach to training by simulating real-life caregiving scenarios in a risk-free environment. Prior to implementation, the environments and the technique’s feasibility and acceptability must be assessed by the professionals who will use it to train caregivers, establishing a performance benchmark based on experienced health care professionals.

**Objective:**

This study aims to test feasibility and develop exploratory benchmarks and acceptability of immersive VR training for home caregiving tasks, using experienced professionals to establish a reference standard for execution quality.

**Methods:**

This observational study was conducted in health care centers in Andalusia, the Valencian Community, and Madrid (Spain). A structured process was followed, including the identification of key home care tasks, the development of best practice guidelines, creation of immersive VR training materials, and the design of a performance evaluation rubric. Health care professionals (n=75) were recruited using a convenience sampling approach. They performed caregiving tasks in VR, and their performance was recorded and assessed using a standardized rubric, which included 205 predefined errors. Participants also completed a posttraining survey evaluating usability, comprehension, and perceived applicability to real-world caregiving.

**Results:**

A total of 75 professionals participated, completing 257 caregiving simulations in a fully immersive VR environment. A total of 417 errors were identified (417/3142, 13.3% of the maximum number of predefined errors), with a mean average of 5.6 (SD 6.8) errors per participant. The most frequent errors occurred in medication management, insulin administration, diaper changing, broncho aspiration prevention, blood pressure monitoring, and hand hygiene. The perceived usefulness of VR training was rated 8.1 out of 10 points (SD 1.9), with 98.7% (74/75) of the participants stating that the time spent in the simulation was worthwhile and 85.3% (64/75) agreeing that the tasks were appropriately represented.

**Conclusions:**

Immersive VR training for informal caregivers is a feasible and well-accepted approach, demonstrating high perceived usefulness among health care professionals. The study establishes a preliminary benchmark for home caregiving task execution, providing a basis for future research evaluating informal caregivers’ performance and targeted training interventions to enhance patient safety. Further studies are needed to explore the long-term impact of VR training on caregiver competence and home care quality.

## Introduction

### Background

Caring for others is a core component of the human existence. Caring is as old as humanity itself; it is both a feeling and a need. This universal dynamic transcends cultures and contexts, forming the foundation of human connections and communities [1].

By 2030, the global population aged 60 years or older is projected to grow to 1.4 billion, and by 2050, this figure is expected to nearly double itself, reaching 2.1 billion [2]. Approximately 80% of these individuals will have 1 or more chronic conditions [3], and between 20% and 30% will experience some degree of disability [4].

Although the increase in life expectancy in recent years represents an unprecedented success for humanity, it also poses a significant challenge [5,6]: how can welfare states remain sustainable while ensuring health and social care for the entire population? The response in many countries has been to develop home care plans where informal caregivers play a central role, particularly for individuals living in remote or rural areas [7]. For example, on September 7, 2022, the European Care Strategy for Caregivers and Care Receivers was approved [8] to ensure quality, affordable, and accessible care services across European countries. This strategy aims to improve conditions for both care recipients and caregivers, whether professional or informal (such as family members), with a particular focus on enhancing home care support. Spain has also embraced this new approach, aligning with the growing care economy [9,10], through the *Estrategia Estatal para un Nuevo Modelo de Calidad de Cuidados en la Comunidad, Proceso de Desinstitucionalización* [11] [State Strategy for a New Model of Quality Care in the Community and the Deinstitutionalization Process] (2024-2030). Based on a person-centered care approach, these strategies advocate for a transformation in the care model for dependent individuals, promoting deinstitutionalization policies and enabling people to remain in their homes for as long as they choose [12].

### Informal Caregiver Portrait

These caregivers are typically unpaid or engaged in precarious roles, predominantly occupied by women [13]. They provide care within the context of an existing relationship, such as a family member, friend, or neighbor, or are hired through social assistance programs or personal resources, often without formal health care qualifications. Care recipients frequently face various challenges, including disabilities, mental illnesses, chronic noncommunicable diseases (including rare conditions), terminal illnesses, substance use disorders, or age-related frailty. In Spain, nearly 82% of dependent individuals are cared for by family members, 90% of whom are women [14]. Most female caregivers are middle-aged, have limited formal education, receive no salary for their caregiving efforts, and are typically spouses, daughters, or daughters-in-law of the care recipients [5].

Today, caregiving at home has become increasingly demanding and complex, with expectations rising as populations age and caregiving needs for both older adult individuals and children grow [15]. This escalating burden not only impacts caregivers—resulting in emotional, physical, and financial strain—but also affects the health and well-being of care recipients. Evidence indicates that unmet caregiver needs and excessive burdens can directly influence recipient mortality rates [16], underscoring the critical importance of supporting caregivers in their role.

### Patient Safety at Home

Ensuring patient safety in home care is a critical yet underexplored aspect of caregiving, particularly as more individuals choose to age or receive treatment at home. While home environments offer comfort and familiarity, they lack the structured safeguards of health care institutions, increasing the risk of unintentional errors that can compromise patient safety and adversely affect health outcomes. Home care patients face various risks of adverse events, including infections, adverse drug reactions, pressure ulcers, fall-related injuries, poisoning, and choking. The rate of medication errors when care is provided by an informal caregiver at home ranges from 2% to 33% of total medications, depending on the complexity of the dosage and the route of administration [17]. Recent estimates suggest that 2% of these medication errors have serious consequences for patients [18]. The risk increases when the patient has low health literacy, receives inadequate caregiving instructions, is cared for by multiple caregivers, or requires complex care. These factors are common causes of errors [8,9].

Errors in home care often led to additional health care resource use, such as emergency consultations or hospital readmissions, placing additional strain on public health care systems [19]. These preventable issues underscore the need for enhanced caregiver support and training to reduce the associated economic and social burden [18]. However, informal caregivers usually do not receive adequate or standardized training before assuming their caregiving responsibilities. At best, they may receive brief and incomplete instructions, which are rarely tailored to their specific caregiving situation or followed up to ensure comprehension and effective application.

The RealityCare Project [20] was developed to create accessible, cost-effective, and efficient training materials and procedures aimed at equipping informal caregivers with the necessary skills to enhance patient safety at home. Unlike traditional passive learning methods, this project leverages immersive and interactive technologies, such as virtual reality (VR), allowing caregivers to acquire essential competencies through active learning. By tailoring training to the specific needs of care recipients, RealityCare seeks to improve care quality, enhance patient safety, and strengthen caregiver preparedness in a highly personalized manner. This initiative draws on a growing body of literature documenting the use of VR technologies in training health care professionals [21-24]. By integrating these innovative training solutions into informal caregiving, RealityCare aims to bridge the knowledge gap that often leads to errors in home care.

To implement this training effectively, it is essential to develop simulated environments where informal caregivers can practice and acquire skills by replicating real-world caregiving scenarios. As a first step in the development of these training environments, the situations in which informal caregivers are most exposed to risks should be identified. Since caregivers often have difficulties recognizing such risks and acknowledging everyday errors, the input and expertise of health care professionals are particularly valuable. Observing how professionals perform these tasks not only provides a useful benchmark to define the typical margin of error but also highlights which tasks are more complex to execute and therefore require targeted training. By focusing on these critical situations, the design of training scenarios can be better aligned with actual caregiving challenges, theoretically enhancing their effectiveness in preparing caregivers for safe and high-quality home care.

The objective of this study was to develop and evaluate the appropriateness, feasibility, use, and acceptability of VR-based training scenarios and procedures for informal caregivers, drawing on the expertise of health care professionals in tasks where informal caregivers are essential partners in supporting the care of chronically ill and dependent patients. These training environments aim to provide caregivers with a structured, immersive, and interactive learning experience, allowing them to develop the necessary skills to ensure safe and high-quality home care.

## Methods

### Study Design

This observational study followed a structured process to develop and iteratively refine fully immersive VR instructional materials for informal caregivers, conceived from the perspective of health care professionals who will use them in practice.

In a first phase (steps 1 and 2), a list of common home care tasks was compiled based on the most prevalent patient profiles, ensuring that the selected tasks accurately reflected real-world caregiving challenges. This phase also included the development of guidelines for the safe execution of home care tasks, integrating best practices to minimize errors and enhance patient safety. To support caregiver training, instructional materials were designed, allowing informal caregivers to recreate home care tasks in a virtual environment according to the specific needs of care recipients. For example, the scenarios included administering subcutaneous medication, preparing insulin, or safely mobilizing a patient. These materials provide an interactive and engaging learning experience, enabling caregivers to practice essential skills in a risk-free, controlled environment.

**Figure 1:**
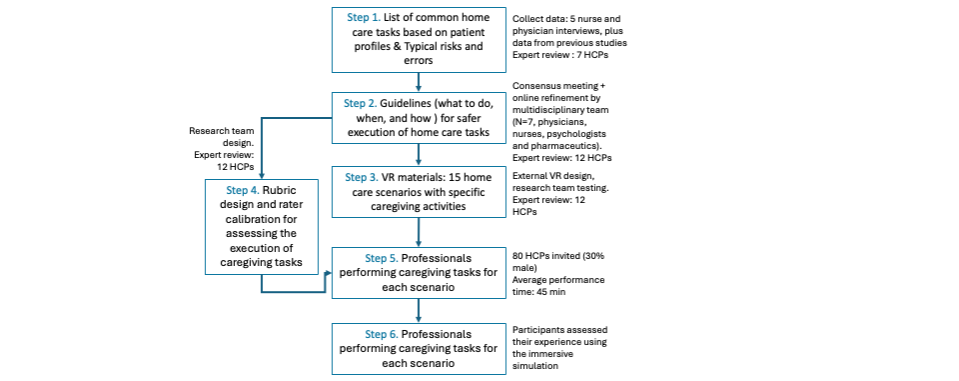
Study flowchart. HCPs: health care providers; VR: virtual reality.

In the second phase (steps 3-6), a rubric for evaluating the execution of home care tasks was then developed, allowing for a structured and consistent assessment of performance and ensuring adherence to best practices. Finally, performance data from a sample of experienced health care professionals were collected within virtual scenarios to establish a reference standard (preliminary benchmark) for home care execution (Figure 1). This benchmark serves as a foundation for future studies assessing informal caregivers’ performance, helping to identify key areas for training improvement.

The main intervention in this study was the performance of caregiving tasks in a fully immersive VR environment. No confounders or effect modifiers were formally controlled in this exploratory study aimed at establishing a preliminary benchmark.

The study was conducted between November 27, 2023, and December 11, 2024, in health care centers located in Andalusia, the Valencian Community, and Madrid.

### Ethical Considerations

The study adhered to ethical principles for research involving human participants, as established in the Declaration of Helsinki. Participation was entirely voluntary and was granted only after obtaining informed consent. The study protocol was approved by the research ethics committee of Hospital de Sant Joan (22/080, February 1, 2023). To ensure privacy and confidentiality, all data were anonymized prior to analysis, and no identifiable information was retained. During the VR sessions, the recordings captured only the participants’ virtual hands, since their faces were fully covered by head-mounted displays (Meta headsets). Participants received a compensation of US $86 for their time.

### Definitions

*Informal caregivers* are those individuals who provide care at home to patients with chronic illnesses, disabilities, or other long-term health or care needs, without having formal qualifications or academic training as health care professionals. These caregivers can be family members or close friends offering support within a personal relationship, but they can also include individuals who are not personally related to the care recipients. This kind of care can be either paid or unpaid. In turn, caregivers who provide professional medical care at home, such as visiting nurses, physiotherapists, social workers, psychologists, or other institutional workers performing medical procedures at home, as well as those offering formal home care assistance services [19], were excluded. *Dependent persons* (recipients) are people with multiple pathologies (a chronic process of more than 2 years of evolution, such as cardiometabolic diseases, respiratory disease, chronic pain, dementia, or reduced mobility) and polypharmacy (using 5 or more drugs daily). *Right care* refers to assisting in the basic and instrumental activities of daily living for persons who require them to promote their well-being meanwhile being safe and respectful of the person’s dignity. *Caregiving error* refers to an avoidable and incorrect action on the part of the caregiver that may or could cause harm to the recipient or provide no benefit to their well-being.

### Setting

The study was conducted in public and publicly contracted health care centers within the Spanish national health system, located in Alicante, Seville, Madrid, and Granada in Spain. The participating centers comprised primary care, home care, and rehabilitation services that regularly interact with informal caregivers, ensuring ecological validity for the evaluation.

### Description of the Study Process

This study followed a structured 6-step process.

#### Step 1: Identification of Common Home Care Tasks

A list of essential caregiving tasks was compiled based on different patient profiles, usually seen in outpatient and primary care consultations, particularly those with chronic conditions or functional dependence. The focus was placed on frequent care activities required in home settings to ensure that the selected tasks accurately reflected real-world caregiving demands. Input was gathered from 5 nurses and physicians working in home care units and primary care services with home assistance, as well as from previous studies [25,26]. This list was then reviewed by 7 health care professionals, who prioritized typical home care scenarios where informal caregivers require specific training to ensure safe care delivery.

#### Step 2: Development of Safe Execution Guidelines

Best practice guidelines were established by a multidisciplinary team (n=7) composed of physicians, nurses, psychologists, and pharmacists with the goal of minimizing errors and enhancing patient safety. The initial content was defined during an in-person consensus meeting and further refined through subsequent web-based exchanges, which allowed the team to specify step-by-step instructions for each task. These guidelines were then reviewed and refined as needed by a panel of 12 nursing and medical professionals with extensive experience in home care for patients with diverse chronic conditions (expert panel on home care).

#### Step 3: Creation of Immersive and Virtual Reality Training Materials

Fully immersive and VR-based instructional content. The first step was the creation of a virtual home environment, complete with different rooms to simulate realistic caregiving settings. In addition, virtual patients were designed, representing both male and female individuals, to reflect diverse care recipients. Once the virtual environment was established, the best practice care guidelines were integrated into the VR system, transforming them into interactive elements within the virtual world. These guidelines were embedded as real-time prompts, decision-making tasks, and scenario-based interventions that caregivers could engage with during training. This approach allows informal caregivers to apply and execute caregiving tasks dynamically, receiving feedback and guidance as they perform each intervention in a risk-free, controlled setting. These scenarios were tested to verify their functionality by 5 members of the research team and by the expert panel on home care, which collaborated in the design of the project's training materials. This procedure also allowed us to ensure the face validity of both the scenarios and the caregiving tasks embedded within them through an iterative qualitative consensus process, whereby every issue raised by the experts was discussed and resolved before the materials were considered finalized. During this process, adjustments were made to address difficulties encountered in navigating the VR environment and to enhance the realism of the scenarios. These refinements ensured that the training materials closely reflected real-world challenges and maximized their relevance and use for subsequent caregiver training. As a result of this development process, a total of 15 scenarios were available for this study. Participants in this step were randomly assigned to the caregiving scenarios they had to perform in a fully immersive environment, serving both as a functionality check and as a means to evaluate performance. Multimedia Appendix 1 includes the scenarios.

#### Step 4: Design of an Evaluation Rubric

Standardized rubrics for each scenario were created to assess the execution of home care tasks, enabling a structured evaluation of caregiver performance. The rubrics were designed by the research team and reviewed by the expert panel on home care, on the basis of errors reported by experienced professionals previously, focusing on those considered most characteristic and clinically relevant, as well as on the most serious risks for patient safety. These errors were listed, hierarchized, and reformulated into clear statements to facilitate consistent understanding and application by evaluators. A total of 205 predefined errors could be coded across the 15 scenarios using the rubrics, representing the range of potential deviations from safe practice that evaluators were instructed to identify. To ensure comprehensiveness and relevance, the rubrics were reviewed by 4 external professionals, not affiliated with the research team, all of whom had substantial experience in patient care and clinical training. It was then tested in practical applications with several cases to confirm that it met the requirements for adequate care delivery in alignment with the study objectives. Three nurses trained in the use of rubrics subsequently applied it to evaluate the performance of participants in this study. Before data collection, nurse reviewers independently coded the same set of randomly selected video recordings; discrepancies were then discussed in a consensus meeting to refine decision rules and exemplars for each error type. Interrater agreement was quantified using Cohen κ. Two calibration cycles were conducted to ensure high consistency in ratings. Multimedia Appendix 2 includes the rubrics used.

#### Step 5: Assessment of Professional Performance in Virtual Scenarios

Experienced health care professionals were enrolled in simulated virtual environment to establish a reference standard (preliminary benchmark) for execution quality. The underlying premise was that the performance of informal caregivers should be assessed against this benchmark, with the expectation that their execution would approximate the standards demonstrated by experienced professionals. All VR sessions were conducted under the supervision of trained personnel to ensure participants’ safety and to prevent accidents. Before starting, participants received a standardized introduction to the VR equipment, including instructions on the use of the head-mounted displays and controllers, and were given a few minutes of practice to familiarize themselves with the environment, taking advantage of the rapid learning curve of the system. From the pool of available scenarios, participants were randomly assigned up to 3, which they were asked to complete by performing caregiving tasks for a virtual patient or by solving problem-based situations, such as the “House of Horrors,” which simulated common challenges in home medication management.

The list of potential errors predefined in the rubric was not disclosed to the participants. As experienced health care professionals, they were already familiar with the correct procedures for each task and were instructed to perform them accordingly within the VR environment. Errors were subsequently identified and coded based on the participants’ performance. An exception was the “House of Horrors” scenario, in which the task explicitly required participants to detect and correct common mistakes typically encountered in home medication management.

The average session lasted approximately 45 minutes, which included an initial period for familiarization with the VR equipment, the virtual environment, and the execution of the designated caregiving tasks. The sessions were recorded by the VR devices. Using standardized rubrics, a primary reviewer assessed each participant’s video recordings; when uncertainty arose, a second reviewer cross-checked the case, and any remaining discrepancies were resolved by consensus. This process ensured that all errors were consistently identified and documented.

#### Step 6. Assessment of the VR Approach by the Participants

Finally, participants evaluated their experience using the immersive simulation through 8 direct questions. The assessment focused on ease of participation, the perceived value of the time spent in the simulation, the attractiveness of the virtual environment, and their ability to adapt to the tool. In addition, participants assessed whether the instructional elements, such as signs and audio, helped them understand the objectives of each scenario, whether the tasks were appropriately represented, and whether the training improved their execution of caregiving tasks. Finally, they evaluated whether the training provided knowledge and skills applicable to their professional practice. The survey was specifically designed by the research team, based on standardized criteria for this type of evaluative instrument [27,28].

### Technical Features of the VR Environment

Participants primarily interacted with the virtual environment using their own hand movements, which were tracked by the head-mounted display to simulate realistic home care situations. Although handheld controllers were available, their use was limited; they were mainly used in specific scenarios, such as the “House of Horrors,” where participants had to point to and select visual responses displayed on the screen. Interaction was restricted to visual and auditory feedback, without additional haptic stimulation.

Real-time prompts were provided during the performance of tasks. Participants also received initial instructions and were expected to carry out caregiving activities autonomously. As an example, in the hand hygiene scenario, participants were asked to open the tap, apply soap, and wash their hands while following the World Health Organization’s Five Moments for Hand Hygiene. On the screen, they received step-by-step visual instructions, and in some cases, a short video demonstration illustrated the correct procedure before they practiced it. This ensured that all participants followed the same standardized sequence across sessions.

The scenarios were designed to allow repeated practice: participants could perform the same sequence as many times as needed until they felt confident that the task had been completed correctly. There was no fixed time limit, but all participants followed identical sequences in each scenario, which ensured comparability and standardization. Sessions were video recorded, but only the care task attempt marked by the participant as valid served as the reference for rubric-based error coding.

### Participants Using Virtual Scenarios

Eighty experienced health care professionals were invited to be involved in this study. This sample size was determined pragmatically, reflecting the exploratory nature of the study, consistent with recommendations for pilot or feasibility studies where sample sizes are often based on practical considerations [29]. Participants were recruited from 10 participating health care centers through an open invitation to staff involved in home visits and in conducting health literacy sessions for informal caregivers. Inclusion criteria required that at least 30% of the sample were male, in line with the gender distribution typically observed in the health care sector. Eligible participants were physicians or nurses with a minimum of 7 years of professional experience and all working in primary care, home care, or units where caregivers are trained to care for patients at home (eg, rehabilitation), with regular contact with patients with chronic conditions. Basic demographic data, including sex, age, years of professional experience, and workplace setting, were collected to ensure that participants met the inclusion criteria and that the sample was balanced in terms of gender distribution. Participants were allocated to scenarios using simple random assignment. Their participation involved using VR devices to perform caregiving tasks for virtual patients, and they were compensated for their time. Multimedia Appendix 3 shows the VR-based environment used.

### Equipment

The VR simulations were run using Meta head–mounted displays. Particularly, Meta Quest 2 headset was used. VR sessions used stand-alone Meta Quest 2 head-mounted displays with inside-out 6DoF tracking and native hand-tracking; controllers were used only when required by a scenario. Headsets were cleaned between uses and fitted with disposable hygiene covers, and all sites used an identical setup to ensure comparability.

### Outcomes

The primary outcome was the number and type of caregiving errors committed by participants during VR scenarios, coded using the standardized rubric. Secondary outcomes included participants perceived usefulness, acceptability, and applicability of VR training, assessed through a postsimulation survey.

### Statistical Analysis

Interrater reliability among reviewers was assessed using Cohen κ to ensure consistency in performance scoring before applying the rubric in the actual study context. The number of errors identified using the rubric was summed, resulting in an error count per participant. Errors were then categorized based on the type of care provided. The rubric evaluations were complete, with no missing data. Descriptive statistics, including totals, means, SDs, percentages, and proportions per scenario, were calculated to summarize participants’ performance across different caregiving tasks. Frequency distributions were used to categorize the number of errors made by the participants. For selected questions on perceived impact, odds ratios (OR) with 95% CIs were calculated based on chi-square 2×2 tables comparing “yes” and “no” responses. The Exact Binomial Test was used to assess the perceived usefulness of the VR training. For these proportions, 95% CIs were calculated using the exact binomial method (Yes rate [95% CI]). These analyses were exploratory, aimed at identifying patterns and trends.

In addition, two complementary analyses were conducted: (1) the 15 caregiving scenarios were grouped into 3 thematic categories for comparison of error rates using chi-square tests and pairwise comparisons with Bonferroni adjustment: administration and medication management (House of Horrors; Subcutaneous Drug Administration, including Insulin, Glucagon, Heparin, Morphine, and Derivatives), daily care (Diaper Change; Daily Hygiene and Correct Selection of Elements in a Hygiene Process; Patient Transfers to Armchair, Bathroom, or Shower from Bed or Wheelchair; Hand Hygiene; Patient with Orthosis; and Care for the Caregiver), and patient monitoring and prevention (Prevention of Bronchial Aspiration in Patients with Dysphagia; Patient with Heart Failure: Weight Changes, Diet Adjustments; Blood Pressure Monitoring; and Prevention of Pressure Ulcers), and (2) professionals were divided into 2 groups according to the number of errors committed (≤3 vs >3) to compare the overall assessment of the VR training received using the Wilcoxon rank sum test with continuity correction. Statistical significance was considered with a *P* value threshold of .05. All statistical analyses were performed using R software (version 4.4.1; R Core Team) [30]. This study was conducted following the STROBE (Strengthening the Reporting of Observational Studies in Epidemiology) [31] guidelines to ensure transparency and quality in reporting observational research.

## Results

### Overview

A total of 75 health care professionals participated in this study (participation rate of 93.7%), collectively performing 257 VR-based caregiving simulations. Meanwhile, 19 out of 75 (25.3%) were males and 56 out of 75 (74.7%) were females. The participants had a mean age of 37.6 (SD 11.5) years. All participants were employed in units with responsibilities that included regular contact with informal caregivers as part of their professional practice. Those who declined to participate did so because they were unable to allocate the necessary time to complete the VR exercise. Interrater reliability among nurse reviewers was high (Cohen κ between 0.88 and 1.00), supporting consistent rubric-based error coding.

A total of 417 errors were recorded, representing 13.3% (417/3142) of the maximum number of predefined errors that could be coded across 257 evaluated scenarios. This corresponds to a mean average of 5.6 errors (SD 6.8) per professional across all tasks. The distribution of errors per scenario is detailed in Table 1.

**Table 1:** Distribution of errors.

Scenario		Number of predefined error types per scenario	Participants	Total observed errors (n), with mean (SD) per participant	Error rate within scenario (% of predefined errors)	Proportion of total errors across all scenarios (% of 417)
**Administration and medication management**		54	84	151 (1.8, 2.5)	3.3	36.2
	Medication errors at home (House of Horrors)	8	41	65 (1.6, 1.4)	19.8	15.6
	Insulin	12	30	73 (2.3, 3.6)	20.3	17.5
	Glucagon	12	6	4 (0.7, 1.6)	5.6	1.0
	Heparin	10	4	6 (1.5, 1.3)	15.0	1.4
	Morphine and derivatives	12	3	3 (1.0, 1.7)	8.3	0.7
**Daily care**		116	126	226 (1.5, 2.8)	1.5	54.2
	Hand hygiene	8	61	60 (1.0, 1.9)	12.3	14.4
	Diaper change	19	31	154 (3.9, 4.1)	26.1	36.9
	Daily hygiene and correct selection of elements in a hygiene process	35	13	9 (0.7, 1.4)	2.0	2.2
	Patient with orthosis	9	8	0 (0.0, 0.0)	0.0	0.0
	Care for the caregiver	17	7	0 (0.0, 0.0)	0.0	0.0
	Patient transfers to armchair, bathroom, or shower from bed or wheelchair	28	6	3 (0.5, 0.8)	1.8	0.7
**Patient monitoring and prevention**		35	47	40 (0.9, 1.0)	2.4	9.6
	Prevention of bronchial aspiration in patients with dysphagia	7	15	11 (0.7, 0.5)	10.5	2.6
	Blood pressure monitoring	10	26	25 (1.0, 1.3)	9.6	6.0
	Patient with heart failure: weight changes, and diet adjustments	11	2	1 (0.5, 0.7)	4.5	0.2
	Prevention of pressure ulcers	7	4	3 (0.8, 1.0)	10.7	0.7
Total		205	257	417 (1.6, 2.5)	13.3	100.0

The highest number of errors, with more than 10 occurrences, was observed in several key caregiving tasks. In medication management at home, frequent mistakes included failing to check medication expiration dates, improper storage, and not ensuring a child-friendly first aid kit. In insulin administration, errors involved inadequate hand hygiene, both in terms of duration and technique. During diaper changing, professionals commonly failed to maintain proper hand hygiene, with errors such as insufficient duration and incorrect maneuvers, rolling the diaper toward the groin instead of away from it, cleaning from the side rather than front to back, and allowing continuous moisture in a specific area. In the prevention of broncho aspiration in patients with dysphagia, one of the main mistakes was positioning the patient facing the food instead of correctly aligning their posture. In blood pressure monitoring, errors were related to incorrect recording and interpretation of average readings between both arms. Finally, in hand hygiene, several professionals neglected essential steps such as rubbing between the fingers in a C-shape, cleaning fingernails by rubbing them against the palm, and washing the thumb properly.

The lowest number of errors recorded was 0, achieved by 18 out of 75 (24.0%) participants, while 57 out of 75 (76.0%) made at least 1 error. At the other extreme, a single professional recorded a maximum of 30 errors. Overall, the distribution showed a higher concentration of participants with few errors, as most made between 0 and 4 mistakes, accounting for 61.3% (46/75) of the total participants. This indicates that most of the participants committed only a small number of errors (Table 2).

**Table 2:** Frequency of errors committed by participants grouped by ranges.

Errors, n	Participants, n	Percentage	Cumulative percentage
0-4	46	61.3	61.3
5-9	17	22.7	84.0
10-14	6	8.0	92.0
15-19	2	2.7	94.7
20-24	3	4.0	98.7
25-30	1	1.3	100.0

Of the 57 professionals who made at least 1 error, 36 out of 57 (63.2%) felt that the simulation improved their execution of the trained task (OR 1.71, 95% CI 1.00-2.94; *P*=.046). In contrast, only 19 out of 57 (33.3%) indicated that the training received applied to their job as well (OR 0.50, 95% CI 0.29-0.87; *P*=.012; Table 3).

**Table 3:** Perceived impact of the simulation on task execution and job applicability for professionals who made errors.

Participants who made at least 1 error (N=57)	Yes, n (%)	No, n (%)	Chi-square (*df*)	*P* value^a^	OR (95% CI)
Did the simulation improve my execution of the trained task?	36 (63.2)	21 (36.8)	3.95 (1)	.046	1.71 (1.00-2.94)
Did I receive training that I can apply to my job?	19 (33.3)	38 (66.7)	6.34 (1)	.01	0.50 (0.29-0.87)

^a^Chi-square test.

The perceived usefulness of this approach was rated with a mean average score of 8.1 out of 10 points (SD 1.9). Table 4 shows the results of the evaluated aspects.

**Table 4:** The perceived usefulness of the virtual reality training.

Evaluated aspect	Yes, n (%)	No, n (%)	Yes rate (95% CI)	*P* value^a^
Was my participation in the study easy?	70 (93.3)	5 (6.7)	84.3%-98.2%	<.001
Was the time dedicated to the simulation worthwhile?	74 (98.7)	1 (1.3)	92.8%-99.9%	<.001
Did I find the simulation attractive?	75 (100)	0 (0.0)	95.2%-100%	N/A^b^
Was I able to adapt to the tool?	65 (86.7)	10 (13.3)	77.9%-92.2%	<.001
Did I understand the objective of each situation thanks to the signs and audio?	59 (78.7)	16 (21.3)	67.5%-87.5%	<.001
Did I see the tasks appropriately represented in the simulation?	64 (85.3)	11 (14.7)	75.6%-92.0%	<.001
Did the simulation improve my execution of the trained task?	46 (61.3)	29 (38.7)	49.9%-72.6%	.06
Did I receive training that I can apply to my job?	53 (70.7)	22 (29.3)	59.2%-80.9%	.001

^a^Exact Binomial Test.

^b^N/A: not applicable.

### Comparison of Error Rates by Scenario Type

When the scenarios were grouped into 3 thematic categories (administration and management of medication, daily care, and patient monitoring or prevention), significant differences were observed in the error rate relative to the total possible errors (χ²_2_=24.01; *P*<.001). After applying pairwise comparisons, the medication management category showed a higher proportion of errors compared with both daily care (*P*<.001) and patient monitoring or prevention (*P*<.001), while no significant differences were found between the latter 2 (*P*=.60; Table 5).

**Table 5:** Error rate by type of scenario (chi-square *P*<.001).

Scenario type	Possible errors	Observed errors	Error rate, %
Administration and medication management	836	151	18.1
Daily care	1883	226	12.0
Patient monitoring and prevention	415	40	9.6
Total	3134	417	13.3

### Perceived Usefulness and Error Burden

**Figure 2:**
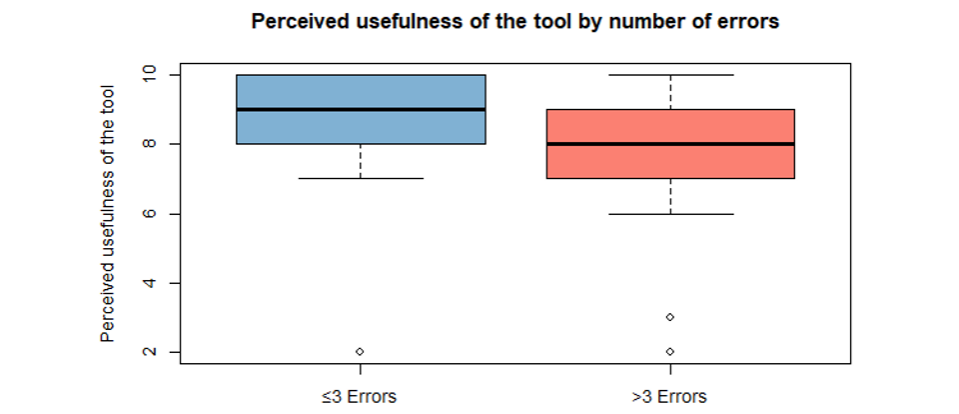
Perceived usefulness of the tool according to number of errors committed.

We compared overall usefulness ratings between professionals with ≤3 errors and those with >3 errors (Wilcoxon rank sum test). No statistically significant difference was observed (*W*=830; *P*=.09). However, the lower-error group tended to rate usefulness higher (mean 8.53, SD 1.97; median 9) than the higher-error group (mean 7.84, SD 1.72; median 8; Table 6). The distribution of perceived usefulness scores according to the number of errors committed is illustrated in the boxplot (Figure 2).

**Table 6:** Overall usefulness ratings by error burden (*P*=.09 determined using the Wilcoxon rank sum test with continuity correction).

Professional group	N	Mean (SD)	Median (IQR)
≤3 errors	45	8.53 (1.97)	9 (8-10)
>3 errors	30	7.84 (1.72)	8 (7-9)

## Discussion

### Principal Findings

This study documents the design, development, and appraisal of immersive educational materials aimed at enhancing the patient safety competencies of informal caregivers in home settings, which health care professionals could use to reduce patient safety risks when care is provided by nonprofessional caregivers (eg, family members). These materials address the patient safety challenges associated with the complex clinical profiles and care needs of care recipients at home, aligning with public policies promoting active aging at home and the growing care economy [32,33].

This approach enabled us to establish preliminary performance standards, providing a reference for evaluating informal caregivers in future studies. It also informed the development of training materials through realistic, evidence-based scenarios.

The materials developed constitute a preliminary library of VR resources. These professional-derived benchmarks and scenarios may inform the future design and evaluation of training programs for informal caregivers; direct testing with informal caregivers is required. There was also a trend whereby participants who perceived that they performed better on the task rated the VR training more highly. Although these findings are relevant, replication in larger and more diverse samples is required before they can be generalized.

These results are consistent with previous studies suggesting some benefits of VR in caregiver education, particularly in improving confidence and competence in real-world caregiving scenarios [34,35]. In line with this, simulation-based training programs have been shown to strengthen caregivers’ confidence and perceived competence in carrying out essential care activities [36]. Participants also tend to regard these programs as practical and beneficial, which reinforces their overall acceptability [37].

### Key Findings and Implications

This study highlights that human error is unavoidable, even among trained professionals performing basic caregiving tasks. Recurring mistakes, especially around the WHO Five Moments for Hand Hygiene [38], reflect persistently suboptimal compliance that can improve with training [39-41].

The study findings indicate a high level of acceptability of the training program among health care professionals, reinforcing the feasibility of implementing VR-based learning tools in caregiver education. However, the scenarios designed for informal caregivers did not always present the expected personal challenges, which may have limited the activation of deeper learning processes. This makes one of the key lessons learned from this study the necessity of incorporating progressive difficulty levels or adaptive challenges to enhance engagement and knowledge retention.

The RealityCare approach focuses on improving patient safety under the care of informal caregivers, addressing both care recipient outcomes and caregiver well-being [42]. In this sense, it is also important to recognize that not all errors have the same implications for patient safety. For instance, medication errors carry a higher risk of severe adverse outcomes than errors in tasks such as diaper changing, which primarily affect patient comfort and dignity. Addressing both dimensions is essential for designing meaningful VR training scenarios that reflect real-world caregiving priorities.

In parallel with the impact that errors may have on patients, these events also increase the emotional burden on caregivers in ways that have received little attention to date. This burden stems from the sense of responsibility associated with making a mistake that directly affects the person under their care—particularly when, as is the case in the majority of situations, a close family relationship is involved. It highlights the need for structured training to reduce stress and mitigate the risk of caregivers experiencing the “double victim” phenomenon [43], where the emotional burden of making mistakes in care impacts both the caregiver and the patient. This aligns with previous studies emphasizing that empowering caregivers with structured training can reduce emotional distress and enhance caregiving quality [44,45].

Another major area of concern was medication management in home care, where frequent errors were observed in tasks such as checking expiration dates, proper storage, and safe administration techniques [46]. Previous studies have shown that medication errors in home settings occur more frequently than assumed, often due to low health literacy, lack of training, and inconsistent caregiving instructions [18,47]. This reinforces the need for targeted training interventions, including problem-solving VR scenarios, where caregivers are required to navigate medication safety challenges to improve their decision-making skills.

### The Role of VR in Caregiver Education

New technologies, such as VR, may be reshaping caregiver education by shifting from passive to active learning approaches [48,49]. Studies on VR training for health care professionals suggest that interactive and immersive simulations may enhance skill acquisition and confidence levels [50]. This study is consistent with recent research indicating that training programs integrating disease-specific information, caregiving techniques, and emotional support strategies enable caregivers to deliver higher-quality care without compromising their well-being [51-53].

### Practical Implications

VR–based simulation is increasingly applied across multiple sectors [54], particularly in health care [55,56]. Although the experience with non–health care professional profiles (including informal caregivers) is still limited, the potential of this approach to enhance caregiving skills is considerable and applicable across a wide range of contexts [57-60]. These data suggest that VR’s impact varies by adaptive scenario design, structured debriefing, progressive difficulty, real-time (including haptic) feedback, appropriate session frequency, curricular integration, cost-effective implementation, and user acceptance [55,56,61-63]. Future work should test whether VR training improves performance on familiar caregiving tasks and addresses navigation or realism issues through the same optimizations to enhance effectiveness and acceptability.

### Limitations and Challenges

Several limitations should be considered when interpreting these findings. First, the generalizability of the findings may be constrained by the recruitment strategy, since health care professionals with relevant responsibilities were invited to participate through a nonrandom, convenience approach. Second, training duration varied according to participants’ learning curves, which may have influenced overall performance. In addition, prior familiarity with digital devices was not controlled, potentially affecting the ease of use of VR technologies among participants. Third, several measures were taken to minimize potential bias. Selection bias was reduced by recruiting physicians and nurses from multiple centers with a balanced profile of sex and experience. Evaluation bias was addressed through a standardized rubric, independent external reviewers, and interrater reliability checks. All participants received brief familiarization with the VR equipment to limit bias related to digital literacy. Nevertheless, residual bias cannot be completely ruled out and should be considered when interpreting results. Another limitation relates to the random assignment of caregiving scenarios, which may have resulted in participants encountering tasks outside their primary area of expertise, leading to higher error rates in unfamiliar situations. Moreover, feedback on the tool itself was collected using yes/no questions rather than Likert-type scales, which may have limited the granularity and nuance of participants’ responses.

In addition, some errors may reflect limitations of the VR environment itself. The immersive environment used may not have fully replicated real-world handling. This factor could have affected user interaction and performance, introducing a source of error that is independent of the participants’ actual caregiving competence.

Future studies should stratify by prior caregiving experience, compare informal caregivers against the professional benchmark established here, and include longitudinal designs to assess real-world outcomes and patient safety events. In addition, personalized VR—using adaptive difficulty, real-time (including haptic) feedback, and artificial intelligence–supported performance tracking—should be evaluated to enhance learning effectiveness and engagement.

The choice to include health care professionals in this first phase was deliberate, as their involvement was essential to validate the materials, ensure that the scenarios were feasible, and provide a preliminary benchmark against which informal caregivers’ performance can later be evaluated. Moreover, professionals are the ones who will ultimately organize and deliver training; without their endorsement and willingness to implement the program, it would not reach informal caregivers. Nevertheless, future studies must directly involve informal caregivers, who are the ultimate target of the intervention and whose perceptions of usefulness, acceptability, and engagement are crucial for its effectiveness. This study therefore represents the necessary first stage in a sequential process, with subsequent research focusing on informal caregivers as the direct trainees.

Finally, these results should not be directly transferred to the training of informal caregivers without adaptation. Unlike professionals, informal caregivers will require detailed instructions to compensate for their lack of prior training and experience, which may influence the applicability and effectiveness of VR-based training.

### Conclusions

This study indicates that immersive VR training is feasible and acceptable among health care professionals, representing an initial step toward implementation and opening the way for future testing with informal caregivers. The use of VR-based simulations to establish a preliminary benchmark for execution quality allows for a systematic assessment of caregiving performance, using experienced health care professionals as a benchmark. By defining realistic, evidence-based expectations for home caregiving tasks, these findings lay the groundwork for future research evaluating the performance of informal caregivers.

VR-based caregiver training has the potential to bridge the knowledge gap in home care, offering safe, structured, and engaging learning experiences. However, further research is required to validate its long-term impact on caregiving competence, patient safety, and informal caregiver well-being.

## Data Availability

The data from this study will be shared upon receiving a reasonable request.

## Appendix

Multimedia Appendix 1 Caregiving scenarios.

Multimedia Appendix 2 Example of rubric.

Multimedia Appendix 3 Virtual reality environment (examples).

## Abbreviations

OR: odds ratio
STROBE: Strengthening the Reporting of Observational Studies in Epidemiology
VR: virtual reality
