# Supplementary material for: Performance and Perceptions of Health Care Professionals Using an Immersive Virtual Reality Tool for Home Care Training: Observational Feasibility and Acceptability Study
Source: JMIR Serious Games. 2025 Nov 20;13:e75104. doi: 10.2196/75104 (PMC12679075; doi:10.2196/75104)
Supplement: Multimedia Appendix 1 [file games_v13i1e75104_app1.docx]

| **Title** | **Description** |
| --- | --- |
| House of Horrors | Identification of typical medication management errors in the home that the caregiver identifies by visiting a virtual home. |
| Subcutaneous Drug Administration: Insulin | Inadequate dosage, hygiene, incorrect location, drug storage. Demonstrate the use of the glucometer. |
| Subcutaneous Drug Administration: Glucagon | Inadequate dosage, hygiene, incorrect location, drug storage. Demonstrate the use of the glucometer. |
| Subcutaneous Drug Administration: Heparin | Inadequate dosage, hygiene, incorrect location, drug storage. |
| Subcutaneous Drug Administration: Morphine and Derivatives | Subcutaneous drug delivery in palliative care. |
| Diaper Change | Do not forget to perform proper hand hygiene beforehand to prevent the patient from developing a urinary tract infection. Maneuvers for the correct performance of this function can also be addressed. |
| Daily Hygiene and Correct Selection of Elements in a Hygiene Process | The elements for the toilet of a person with reduced mobility, bedridden or with some ability to move are presented. The sequence is from the beginning so that all the elements have to be chosen. |
| Prevention of Bronchial Aspiration in Patients with Dysphagia | Correct oral hygiene to reduce bacterial colonization in the oropharynx, Positioning of the patient for eating, rhythm, environmental conditioning factors. Management of feeding textures. Warning signs. |
| Patient Transfers to Armchair, Bathroom, or Shower from Bed or Wheelchair | Falls, bumps, scratches, picking it up from inappropriate parts, coming into contact with its dressings, ostomies, wounds, etc. |
| Patient with Heart Failure: Weight Changes, Diet Adjustments | Management of situations of weight gain due to oedema, other symptoms such as shortness of breath, swelling, dry cough. Blood pressure measurement, use of a diuretic to regulate oedema. |
| Blood Pressure Monitoring | Taking blood pressure safely. |
| Prevention of Pressure Ulcers | Maneuvers and massages to check that the area is well vascularized, strategies to see if a possible injury is a bedsore or not, pressure relief of the area. Review of areas prone to developing a bedsore. |
| Hand Hygiene | The person follows all movements of the OMS and cleans all surfaces of the hand. The person should stay long enough on each part of the hand, and should not go faster than he/she touches. |
| Patient with Orthosis | Use of orthoses if a clubfoot is present. |
| Care for the Caregiver | Prevention of injuries and damage to the back and joints due to bad positions or poor execution of movements. |

Supplementary Materials. List of scenarios
