# Supplementary material for: Performance and Perceptions of Health Care Professionals Using an Immersive Virtual Reality Tool for Home Care Training: Observational Feasibility and Acceptability Study
Source: JMIR Serious Games. 2025 Nov 20;13:e75104. doi: 10.2196/75104 (PMC12679075; doi:10.2196/75104)
Supplement: Multimedia Appendix 2 [file games_v13i1e75104_app2.docx]

| Hand Hygiene | Action performed incorrectly |
| --- | --- |
| The person opens the water tap and wets their hands |  |
| Soap is applied |  |
| Palms of the hands are rubbed against each other |  |
| Then the back of both hands is rubbed |  |
| Soap is applied between the fingers of both hands |  |
| Fingers are rubbed cleaning the inside, forming a C shape |  |
| Nails are cleaned by rubbing them against the palm of the hand |  |
| The thumb is washed |  |
| Total number of errors |  |
